# Supplementary material for: A negative feedback loop between JNK-associated leucine zipper protein and TGF-β1 regulates kidney fibrosis
Source: Commun Biol. 2020 Jun 5;3:288. doi: 10.1038/s42003-020-1008-z (PMC7275040; doi:10.1038/s42003-020-1008-z)
Supplement: Supplementary file 1 — Supplementary Information [file 42003_2020_1008_MOESM1_ESM.pdf]

- ## Supplementary Figures

Supplementary Figure 1. Generation of conditional Jlp knockout (KO) mice.

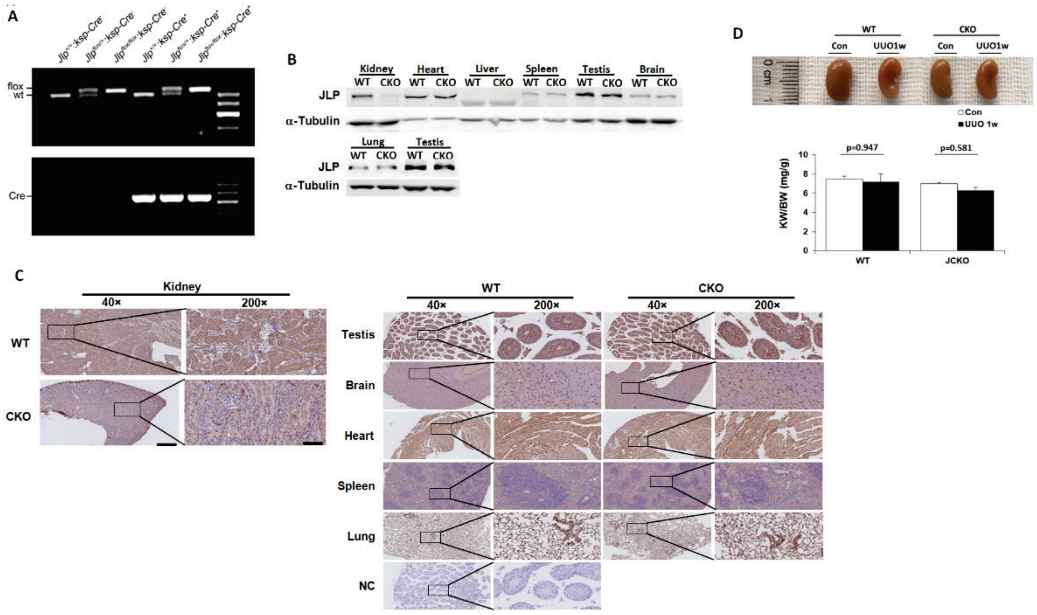

(A) Genotyping serial genotypic mice including the TECs-specific Jlp KO mice. Jlp protein expressing in renal and other tissues of indicated genotypic mice were detected by western blot (B) or immnofuorescence staining (C). (D) Comparison between kidneys of wildtype and Jlp-cKO mice in the aspects of morphological appearance and weight. n=3. SD are marked by error bars.

Supplementary Figure 2. Generation of conditional Jlp transgenic (TG) mice.

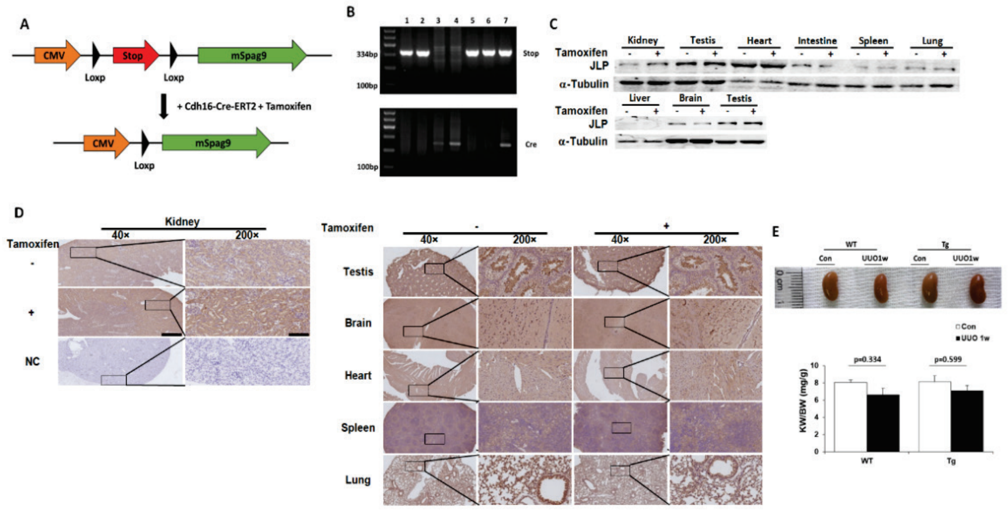

(A) Diagram of conditional mSpag9 transgene. The CMV drives Spag9 expression from this transgene after removal of a loxp-flanked STOP cassette by Cdh16-Cre recombinase. (B) Genotypes of conditional mSpag9 TG mice were confirmed by PCR. STOP allele shows 334 bp sized band, and Cre allele 257 bp. One STOP/Cre mice (#7), four STOP mice (#1, #2, #5, #6) and two Cre mice (#3, #4) are shown. Jlp protein expressing in renal and other tissues of indicated genotypic mice were detected by western blot (C) or immunofluorescence staining (D). (E) Comparison between kidneys of wildtype and Jlp-cKO mice in the aspects of morphological appearance and weight. n=3. SD are marked by error bars.

Supplementary Figure 3. Scaffold protein Jlp deficiency in renal intrinsic cells was involved in UUO-induced kidney fibrosis.

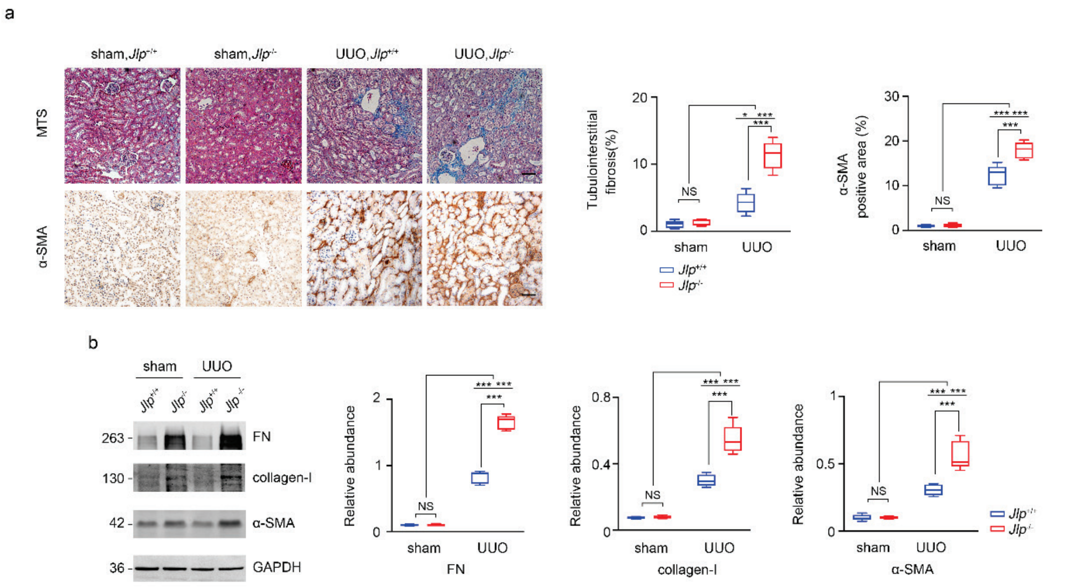

Jlp$^{+/+}$ and Jlp$^{-/-}$ mice were irradiated with X-ray to deplete bone marrow cells and then transfused with congenetic normal bone marrow, followed by UUO treatment. (a) Representative images of MTS staining and IH staining of α-SMA in the renal cortex from the indicated groups. Scale bar, 50μm. (b) Quantification of percentage of interstitial fibrosis of kidney from indicated groups. Mean±SDs were calculated from three visual fields for each kidney sample. n=5. * P<0.05. (c) Quantification of IHC intensity of α-SMA from the different groups as indicated. Mean±SDs were calculated from three visual fields for each kidney sample. SD are marked by error bars, * P<0.05. (d) Western blotting analyses showing the expression of TGF-β, FN and α-SMA in sham-operation kidney samples and kidney samples with UUO from Jlp +/+ and Jlp -/- mice. GAPDH sets as loading control. (e-g) Relative abundance of TGF-β (e)、FN (f) and α-SMA (g) expression was normalized by the abundance of GAPDH, Mean±SDs were calculated from three independent experiments. SD are marked by error bars, NS = no significant difference, * P<0.05, ** P<0.01, *** P<0.001.

Supplementary Figure 4. Jlp is predominated expressed in renal epithelial cells.

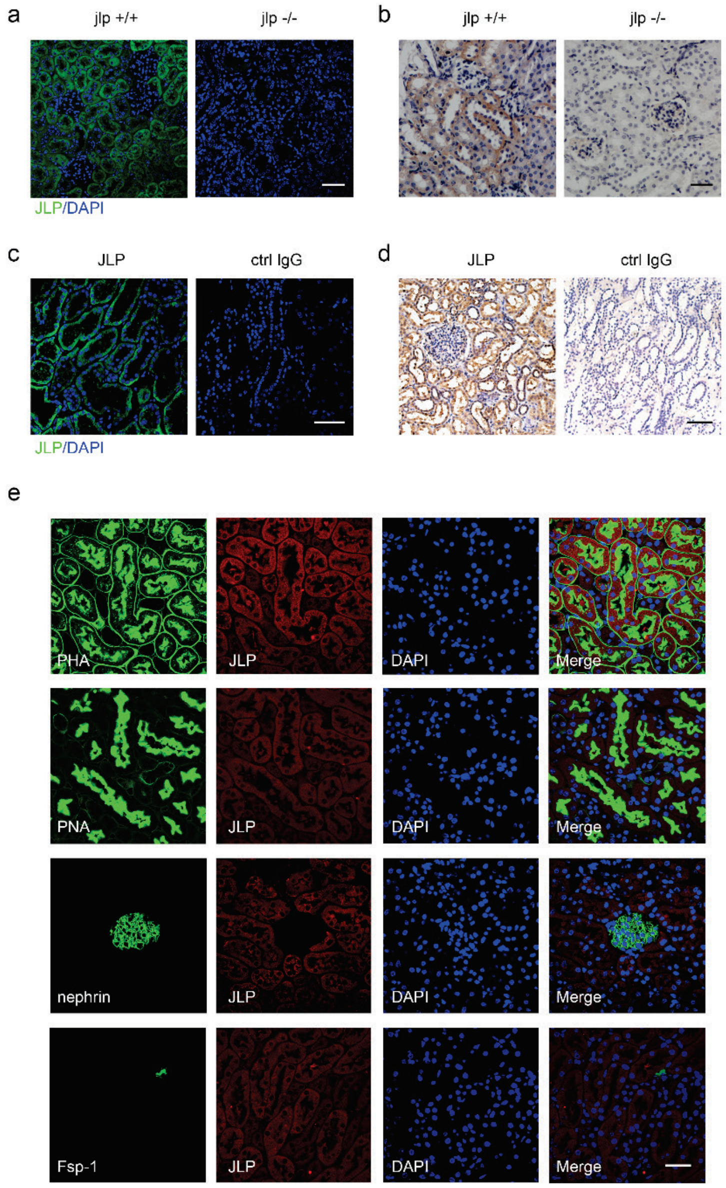

(a) Representative images of IF staining of Jlp (green) in kidney from Jlp +/+ and Jlp -/- mice. Scale bars, 50μm. (b) Representative images of immunohistochemical staining of Jlp in kidney from Jlp +/+ and Jlp -/- mice. Scale bars, 50μm. (c) Representative images of IF staining of Jlp (green) in normal human kidney samples (n=4). Scale bars, 10μm. (d) Representative images of immunohistochemical staining of Jlp in normal human kidney samples (n=4). Scale bars, 20μm. (e) Representative images of IF double staining of Jlp (red) and PHA (marker of proximal TECs), Jlp and PNA (marker of distal TECs), Jlp and Nephrin (marker of glomerulus), as well as Jlp and Fsp-1 (marker of

renal interstitial fibroblasts) in kidney from Jlp +/+ mice. Cell nuclei were visualized with DAPI co-staining. Scale bars, 50μm.

Supplementary Figure 5. TECs specific transgenic expression of Jlp mitigated UUO induced autophagy activation.

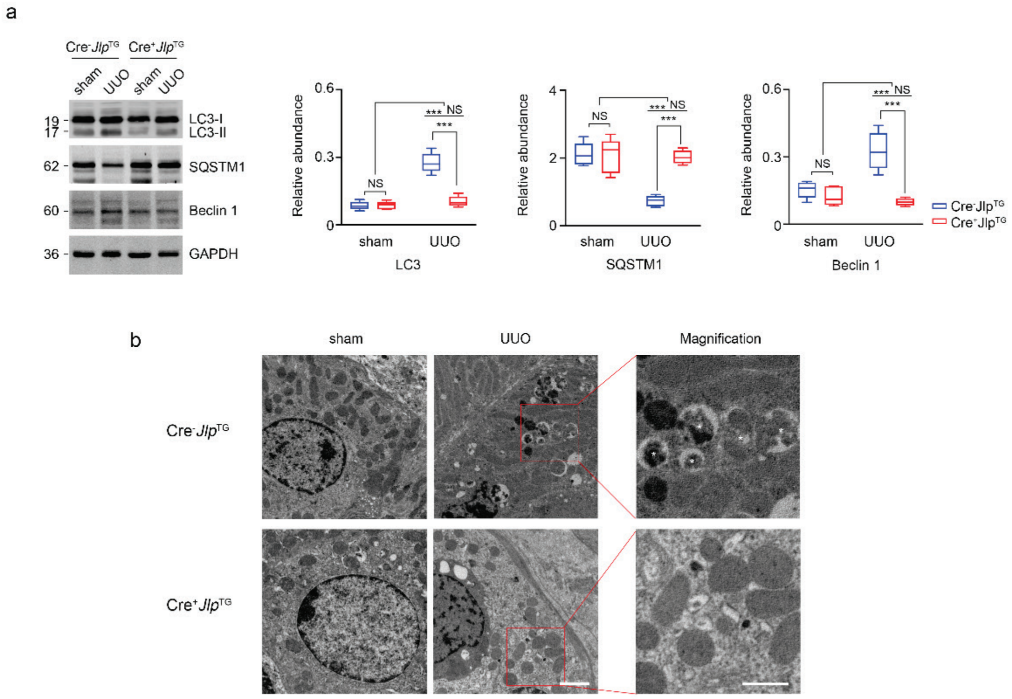

(a) Western blotting analyzing the expression of LC3, SQSTM1, and Beclin-1 protein in kidneys from indicated experimental groups (left panel). Relative abundance of LC3, SQSTM1, and Beclin-1 expression were normalized by the relative abundance of GAPDH, GAPDH was set as loading control. Mean ± SDs were calculated from three independent experiments. n=5. NS = no significant difference, * P<0.05, ** P<0.01, *** P<0.001. (b) Electron microscopic images of autophagic vacuoles in TECs from indicated groups. White asterisks indicate autophagic vacuoles. Scale bars, 2μm. (insets, 1μm).

Supplementary Figure 6. Schematic illustration of Jlp in counteracting the TGF-β1 induced profibrotic effects on TECs.

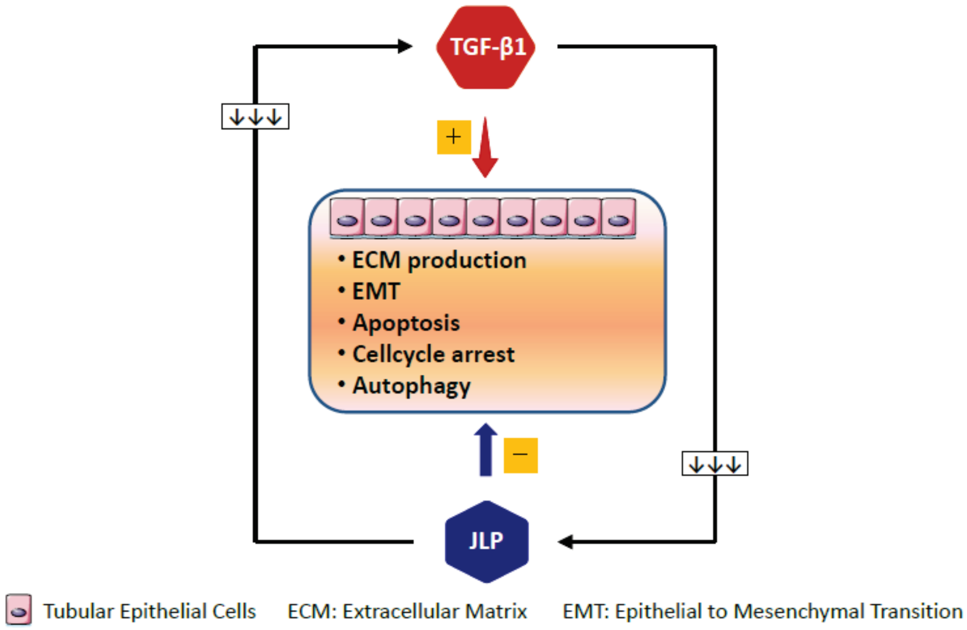

TGF-β1 elicits the effects of ECM production, EMT, apoptosis, cell cycle arrest, and autophagy on TECs, thus promote renal fibrosis, whereas thesffects of TGF-β1 could be antagonized by Jlp. In addition, there is a negative feedback loop exist between Jlp and TGF-β1 on their expression in TECs.

Supplementary Figure 7

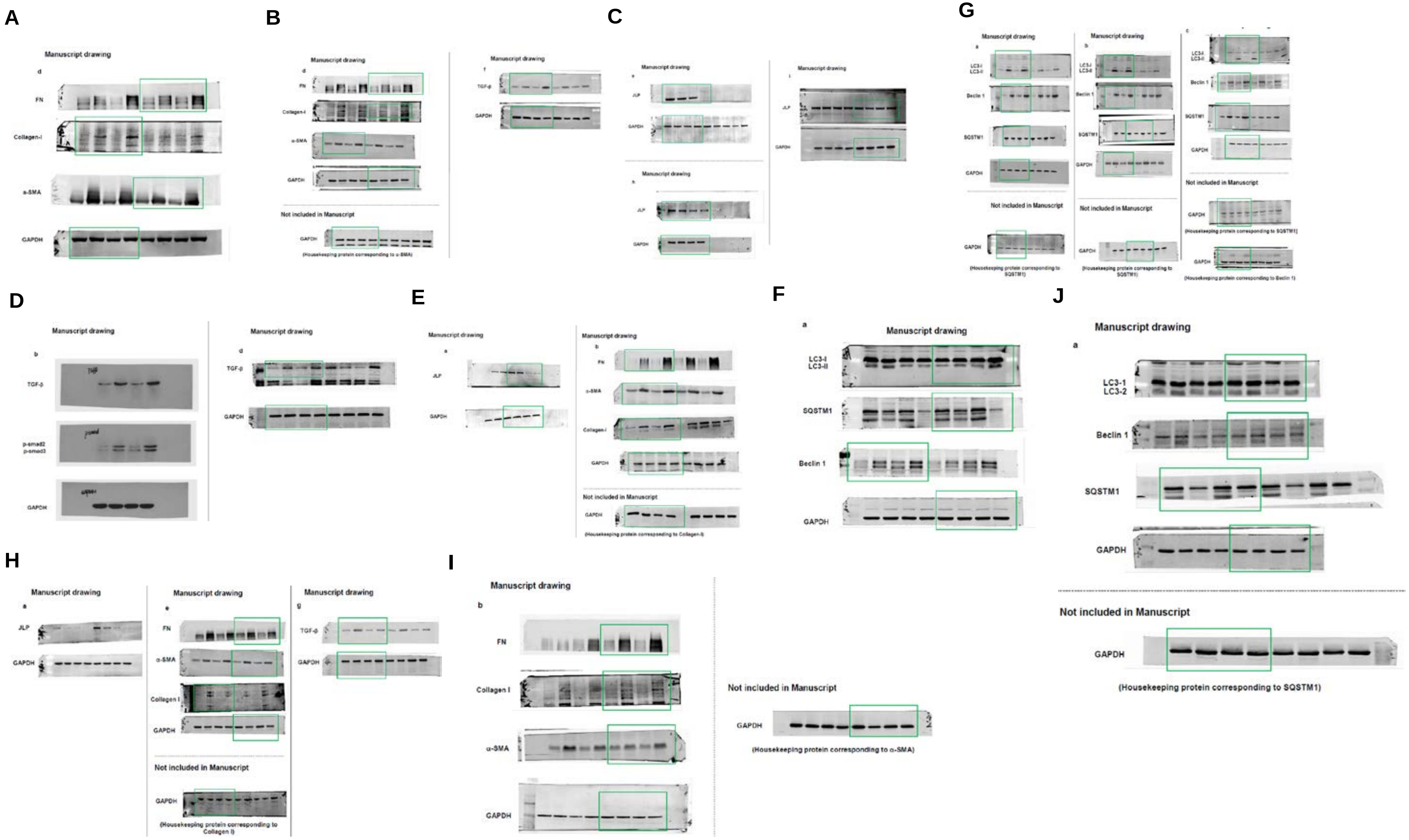

(A) WB band full image of Fig 1. (B) WB band full image of Fig 2. (C) WB band full image of Fig 3. (D) WB band full image of Fig 4. (E) WB band full image of Fig 5. (F) WB band full image of Fig 6. (G) WB band full image of Fig 7. (H) WB band full image of Fig 8. (I) WB band full image of Supplementary figure 3. (J) WB band full image of Supplementary figure 5.

- **Supplementary tables**

Supplementary Table 1. Sequences of oligonucleotide primers used for qPCR.

Gene	Forward	Reverse
Hu JLP	CAGTAAACAGCGAAGTGCC	TTTGTAGCCGAATGAGTGC
Hu actin	AGCGAGCATCCCCCAAAGTT	GGGCACGAAGGCTCATCATT
Mus JLP	GTAACCGTCCTGGAAGTGTAA	CTGCTACTTTGTGGGCTAATG
Mus TGF	TTGCTTCAGCTCCACAGAGA	TGGTTGTAGAGGGCAAGGAC
Mus FN	ACCAGTTGGCACCGACGAAGAGC	GCGTAATGGGAAACCGTGTAAGG
Mus Col-I	GGCAAAGATGGAGAAGCTGG	GGAAACCTCTCTCGCCTCTT
Mus a-SMA	GACCCAGATTATGTTTGAGACC	CTCCAGAGTCCAGCACAATACCA
Mus GAPDH	AAGAGGGATGCTGCCCTTAC	ATCCGTTCACACCGACCTTC

Supplementary Table 2. Clinical characteristics of patients with CKD and normal controls.

	No.	Gender	Age	Diagnosis	BUN(mg/dl)	Serum Cr(mg/dl)	eGFR(ml/min/1.73m^2)
Normal controls (n=4)	1	F	48	IgA nephropathy	16.6	1.1	89.2
	2	F	26	IgA nephropathy	14.3	1.3	91.4
	3	M	19	MCNS	12.2	0.9	113.4
	4	F	36	MN	17.3	0.8	102.7
CKD patients (n=4)	5	F	53	Diabetic nephropathy	36.7	4.5	30.2
	6	M	24	IgA nephropathy	50.2	6.6	16.8
	7	F	37	FSGS	42.9	5.7	28.4
	8	F	49	Hypertensive-induced TIF	48.1	6.4	18.8

Clinical characteristics of patients with CKD and normal controls (n-4, respectively) are shown. CKD: chronic kidney diseases, MCNS: minimal change nephrotic syndrome, MN: membranous nephropathy, FSGS: focal segmental glomerular sclerosis, TIF: tubulointerstitial fibrosis, BUN: blood urea nitrogen, Cr: creatinine, eGFR: etimated glomerular filtration rate.
