# Supplementary material for: A negative feedback loop between JNK-associated leucine zipper protein and TGF-β1 regulates kidney fibrosis
Source: Commun Biol. 2020 Jun 5;3:288. doi: 10.1038/s42003-020-1008-z (PMC7275040; doi:10.1038/s42003-020-1008-z)
Supplement: Supplementary file 2 — Description of Additional Supplementary Files [file 42003_2020_1008_MOESM2_ESM.pdf]

Description of Additional Supplementary Files

File Name: **Supplementary Data 1**
Description: Source data underlying the relevant graphs and charts in the figures.
