# Supplementary material for: A negative feedback loop between JNK-associated leucine zipper protein and TGF-β1 regulates kidney fibrosis
Source: Commun Biol. 2020 Jun 5;3:288. doi: 10.1038/s42003-020-1008-z (PMC7275040; doi:10.1038/s42003-020-1008-z)
Supplement: Supplementary file 4 — Reporting Summary [file 42003_2020_1008_MOESM4_ESM.pdf]

nature research

Corresponding author(s): Huiming Wang

Last updated by author(s): Apr 25, 2020

Reporting Summary

Nature Research wishes to improve the reproducibility of the work that we publish. This form provides structure for consistency and transparency in reporting. For further information on Nature Research policies, see Authors & Referees and the Editorial Policy Checklist.

Statistics

For all statistical analyses, confirm that the following items are present in the figure legend, table legend, main text, or Methods section.

n/a	Confirmed	
☐	☒	The exact sample size (n) for each experimental group/condition, given as a discrete number and unit of measurement
☐	☒	A statement on whether measurements were taken from distinct samples or whether the same sample was measured repeatedly
☐	☒	The statistical test(s) used AND whether they are one- or two-sided<br>*Only common tests should be described solely by name; describe more complex techniques in the Methods section.*
☐	☒	A description of all covariates tested
☐	☒	A description of any assumptions or corrections, such as tests of normality and adjustment for multiple comparisons
☐	☒	A full description of the statistical parameters including central tendency (e.g. means) or other basic estimates (e.g. regression coefficient) AND variation (e.g. standard deviation) or associated estimates of uncertainty (e.g. confidence intervals)
☐	☒	For null hypothesis testing, the test statistic (e.g. F, t, r) with confidence intervals, effect sizes, degrees of freedom and P value noted<br>*Give P values as exact values whenever suitable.*
☒	☐	For Bayesian analysis, information on the choice of priors and Markov chain Monte Carlo settings
☒	☐	For hierarchical and complex designs, identification of the appropriate level for tests and full reporting of outcomes
☒	☐	Estimates of effect sizes (e.g. Cohen's d, Pearson's r), indicating how they were calculated

Our web collection on statistics for biologists contains articles on many of the points above.

Software and code

Policy information about availability of computer code

Data collection	No software was used.
Data analysis	Odyssey CLX Infrared Image System (v3.0), Quantity One (v4.6.9), Image J (v1.37c), Flow Jo (v10.0), SPSS (v17.0) GraphPad Prism (v6.0),FV1200 viewer (v4.0), ABI7900 SDS (v2.4).

For manuscripts utilizing custom algorithms or software that are central to the research but not yet described in published literature, software must be made available to editors/reviewers. We strongly encourage code deposition in a community repository (e.g. GitHub). See the Nature Research guidelines for submitting code & software for further information.

Data

Policy information about availability of data

All manuscripts must include a data availability statement. This statement should provide the following information, where applicable:

- Accession codes, unique identifiers, or web links for publicly available datasets
- A list of figures that have associated raw data
- A description of any restrictions on data availability

All data that support the findings of this study are available from the corresponding author upon reasonable request.

Field-specific reporting

Please select the one below that is the best fit for your research. If you are not sure, read the appropriate sections before making your selection.

☒ Life sciences ☐ Behavioural & social sciences ☐ Ecological, evolutionary & environmental sciences

Life sciences study design

All studies must disclose on these points even when the disclosure is negative.

Sample size	For human study, 4 sample for chronic kidney diseases patients and 4 sample for healthy control were used.<br>For animal study, a sample size of at least 5 mice per group were determined based on the means and variation of published experiments(PMID: 30062051). We routinely exceeded the minimal number required for each experiments as indicated in the figure legends.
Data exclusions	No data were excluded from the analyses.
Replication	All attempts at replication were successful.
Randomization	Randomization was not used in human sample collection.<br>Mice were randomly distributed prior to UUO surgery.
Blinding	Blinding was not used in human sample collection. Staining images were evaluated by pathologists who were blinded to other data. Investigators were blinded to allocation during experiments and outcome assessments.

Reporting for specific materials, systems and methods

We require information from authors about some types of materials, experimental systems and methods used in many studies. Here, indicate whether each material, system or method listed is relevant to your study. If you are not sure if a list item applies to your research, read the appropriate section before selecting a response.

Materials & experimental systems

n/a	Involved in the study	
☐	☒	Antibodies
☐	☒	Eukaryotic cell lines
☒	☐	Palaeontology
☐	☒	Animals and other organisms
☐	☒	Human research participants
☐	☒	Clinical data

Methods

n/a	Involved in the study	
☒	☐	ChIP-seq
☐	☒	Flow cytometry
☒	☐	MRI-based neuroimaging

Antibodies

Antibodies used	Jlp (ab12331), Fsp-1 (ab197896), α-SMA (ab124964), Fibronectin (ab45688), Collagen-I (ab34710), Ki67 (ab16667), TGF-β (ab92486), phospho-Smad2 (ab188334), phospho-Smad3 (ab52903) and p62 (ab56416) were all from Abcam (Cambridge, MA). Anti-LC3 antibody (ab51520, Abcam) was used for immuno-staining and anti- LC3 antibody (L7543, Sigma) was used for western blotting, respectively. Anti- Nephrin and anti-F4/80 (123120) antibodies were from Progen and Biolegend, respectively. Anti-Caspase-3 (#9664), anti-Beclin-1 (#3738) were from Cell signaling Technology (Danvers, MA). Anti-GAPDH (sc-365062) was purchased from Santa Cruz.
Validation	All the antibodies were previously validated by the manufacturer.

Eukaryotic cell lines

Policy information about cell lines

Cell line source(s)	Human TEC cell lines HK-2 was purchased from China Centre for Type Culture Collection.
Authentication	HK-2 cells was validated by China Centre for Type Culture Collection and culture protocol was previously validated in our lab (PMID:30062051)
Mycoplasma contamination	HK-2 cells were not tested for mycoplasma contamination.
Commonly misidentified lines (See ICLAC register)	No commonly misidentified cell lines were used in our study.

Animals and other organisms

Policy information about studies involving animals; ARRIVE guidelines recommended for reporting animal research

Laboratory animals	Jlp Wild type (Jlp+/+) and Jlp global deficient (Jlp-/-) mice were generated by inbreeding Jlp+/- mice in our previously work

Laboratory animals	(PMID:25586186, PMID:28521278). TECs-specific Jlp deficient mice and TEC-specific Jlp transgenic (Tg) mice (all C57/BL6) were generated and purchased from Cyagen Biosciences. Ksp-Cre mice were purchased from Jackson Lab (Stock No: 012237). 8- to 10-week-old male mice were used in this study.
Wild animals	This study did not involve wild animals.
Field-collected samples	This study did not involve samples collected from the field.
Ethics oversight	All animal experiments were approved by the Animal Ethics Review Board of Wuhan University and performed in accordance with the guidelines of the National Health and Medical Research Council of China.

Note that full information on the approval of the study protocol must also be provided in the manuscript.

Human research participants

Policy information about studies involving human research participants

Population characteristics	The detailed information of human participants can be found in Supplementary Table 2.
Recruitment	Kidney samples were obtained from kidney biopsy from renmin hospital of Wuhan University.
Ethics oversight	Human study was approved by medical ethics committee of Wuhan University

Note that full information on the approval of the study protocol must also be provided in the manuscript.

Clinical data

Policy information about clinical studies

All manuscripts should comply with the ICMJE guidelines for publication of clinical research and a completed CONSORT checklist must be included with all submissions.

Clinical trial registration	*Provide the trial registration number from ClinicalTrials.gov or an equivalent agency.*
Study protocol	*Note where the full trial protocol can be accessed OR if not available, explain why.*
Data collection	*Describe the settings and locales of data collection, noting the time periods of recruitment and data collection.*
Outcomes	*Describe how you pre-defined primary and secondary outcome measures and how you assessed these measures.*

Flow Cytometry

Plots

Confirm that:

☐ The axis labels state the marker and fluorochrome used (e.g. CD4-FITC).

☒ The axis scales are clearly visible. Include numbers along axes only for bottom left plot of group (a 'group' is an analysis of identical markers).

☒ All plots are contour plots with outliers or pseudocolor plots.

☒ A numerical value for number of cells or percentage (with statistics) is provided.

Methodology

Sample preparation	Human TEC cell lines HK-2 were cultured and harvested for flow cytometry.
Instrument	BD Accuri C6 was applied for data collection.
Software	FlowJo (v10.0) was used for data analysis.
Cell population abundance	Cultured cell lines were not need to purify cell population.
Gating strategy	Cultured cell lines were not need to gate cell population.

☐ Tick this box to confirm that a figure exemplifying the gating strategy is provided in the Supplementary Information.
